# Protein-Coding Genes of *Helicobacter pylori* Predominantly Present Purifying Selection though Many Membrane Proteins Suffer from Selection Pressure: A Proposal to Analyze Bacterial Pangenomes

**DOI:** 10.3390/genes12030377

**Published:** 2021-03-06

**Authors:** Alejandro Rubio, Antonio J. Pérez-Pulido

**Affiliations:** Centro Andaluz de Biología del Desarrollo (CABD, UPO-CSIC-JA), Facultad de Ciencias Experimentales (Área de Genética), Universidad Pablo de Olavide, 41013 Sevilla, Spain; arubval@upo.es

**Keywords:** *Helicobacter pylori*, genome annotation, purifying selection, outer membrane protein, pangenome

## Abstract

The current availability of complete genome sequences has allowed knowing that bacterial genomes can bear genes not present in the genome of all the strains from a specific species. So, the genes shared by all the strains comprise the core of the species, but the pangenome can be much greater and usually includes genes appearing in one only strain. Once the pangenome of a species is estimated, other studies can be undertaken to generate new knowledge, such as the study of the evolutionary selection for protein-coding genes. Most of the genes of a pangenome are expected to be subject to purifying selection that assures the conservation of function, especially those in the core group. However, some genes can be subject to selection pressure, such as genes involved in virulence that need to escape to the host immune system, which is more common in the accessory group of the pangenome. We analyzed 180 strains of *Helicobacter pylori*, a bacterium that colonizes the gastric mucosa of half the world population and presents a low number of genes (around 1500 in a strain and 3000 in the pangenome). After the estimation of the pangenome, the evolutionary selection for each gene has been calculated, and we found that 85% of them are subject to purifying selection and the remaining genes present some grade of selection pressure. As expected, the latter group is enriched with genes encoding for membrane proteins putatively involved in interaction to host tissues. In addition, this group also presents a high number of uncharacterized genes and genes encoding for putative spurious proteins. It suggests that they could be false positives from the gene finders used for identifying them. All these results propose that this kind of analyses can be useful to validate gene predictions and functionally characterize proteins in complete genomes.

## 1. Introduction

Prediction of genes is usually the first step followed when a new genome is completely sequenced and assembled [1]. The annotation of genes in bacteria is facilitated by the short length of its intergenic sequences and the lack of exon/intron structure. However, computational tools for predicting genes in bacteria fails in around 5% of cases, and the number of uncharacterized genes keeps high levels, especially in little-studied bacteria [2,3].

Missannotation and errors in the annotation of complete genomes can be overcome when a great number of sequences is available, and the species pangenome can be estimated and characterized [4,5]. The comparison of genes coming from different genome sequences of the same or nearby species is useful to validate genes, and to study their evolutionary history [6]. These footprints of the past include conserved amino acids in the same protein from different strains, which reflects purifying selection that maintain both protein structure and function, and amino acid changes in homologous proteins that point to selection pressure, for example, in proteins bearing antigenic regions recognized by the immune system of host organisms [7,8]. The former kind of selection is originating from both invariable or synonymous codons in the coding sequence, and the latter comes from nonsynonymous codons.

The ratio of nonsynonymous (Ka) to synonymous (Ks) substitution rates has been widely used to study the kind of selection occurring in specific genes. A value lower than 1 (Ks > Ka) is expected in the majority of genes in order to conserve their original function, especially when they are essential genes, but the ratio can be greater than 1 (Ks < Ka) when the gene is subjected to positive selection [9,10,11]. To calculate this value, homologous sequences are usually searched and aligned, and the availability of many strains from the same species allows today the intraspecies calculation, which enables one to consider a narrow divergence time. Theoretically, intraspecies Ka/Ks calculation could improve the annotation of complete genomes, highlighting uncharacterized genes with high ratios that could be spurious sequences, or annotating genes as subjected to selective pressure, or even finding alternative open reading frames inside the already annotated ones.

To test this hypothesis, we used a dataset of complete genomes from *Helicobacter pylori*. It is a Gram-negative bacterium that persistently colonizes the gastric mucosa of half the world population, a highly variable environment that is hostile to virtually all other bacterial species [12]. Infections by this bacterium are associated with chronic gastritis, stomach and duodenal ulcers, and even gastric cancer [13]. The genome of any strain of *H. pylori* bears around 1500 genes, with 75% of them shared by all the strains and constituting its core genome [14]. Some of these genes are involved in virulence and are subjected to a strong selection pressure due to their interaction with the host, mainly the immune system that they need to avoid. Many proteins from the bacterium are localized into the membrane and are involved in adherence and pathogenesis [15,16]. Other characteristic structures created by the cells of *H. pylori* are the outer membrane vesicles (OMVs), where the oncoprotein CagA has an important role. OMVs bear various biologically active compounds, which internalize into host cells, and they can affect signaling pathways and promoting apoptosis of gastric epithelial and immunocompetent cells [17,18]. Finally, many proteins encoded by the genome of *H. pylori* are annotated as unknown proteins with uncharacterized functions [2].

When analyzing the rate of nonsynonymous versus synonymous substitutions of genes from the *H. pylori* pangenome, we found that most of them are subject to purifying selection, especially those belonging to the core genome. However, a small number of genes present a higher rate of nonsynonymous changes. Some of them could be genes subject to selective pressure, mainly encoding for membrane proteins that putatively interact with host cells. However, others could be spurious genes erroneously predicted by gene finders.

## 2. Materials and Methods

### 2.1. Genome Sequences, Pangenome Estimation, and Functional Annotation

Complete assembled sequences of the 180 genomes of *H. pylori* stored in the NCBI Genome database until December 2020 were collected (Supplementary Table S1). Protein-coding genes were predicted using Prokka version v1.13.4 [19], and the predicted amino acid sequences were functionally annotated using Sma3s v2 and UniProt taxonomic division bacteria 2018_05 as the reference database [20]. To assess the core and pangenome, Roary version 3.12.0 was used with an identity threshold of 90% and the *-s* parameter for not separating paralogs at this identity threshold [21]. This process creates groups of genes that assume the same gene coming from the different strains, and a reference sequence would represent each group. To be more exhaustive, we used our previous protocol [4], where the reference genes are functionally annotated by Sma3s, and proteins with the same gene name are collapsed. In this way, we had high confidence in the presence/absence of every gene in the pangenome. Finally, we defined 4 different groups regarding the percent of strains that present each gene: core (>99%), accessory (20–99%), cloud (1–20%), and singleton (<1%; 1 strain).

### 2.2. Ratio Ka/Ks Calculation

The software KaKs_Calculator was used to calculate the ratio Ka/Ks [22]. The coding sequence of each protein-coding gene was used as the started input, and BLASTN 2.9.0+ [23] was used to search for homologous sequences in all the available genomic sequences of *H. pylori*. If the similarity search finds homologous sequences, they are aligned together with the starting sequences, using MAFFT version 7.305 [24]. When gaps are not multiples of three in one of the sequences, the missing gaps to reach this multiplicity are added to the end of this sequence, so maintaining each sequence with a multiple length of three and suitable with a complete reading frame. Then, the sequences from these alignments were transformed into the five remaining reading frames by Seqkit software version 0.15 [25]. Finally, the Ka/Ks ratio for the six putative reading frames was calculated using the multiple alignment with KaKs_Calculator 2.0.

### 2.3. Functional Enrichment

The TopGO R package version 2.40.0, which uses GO terms from a specific ontology, was used to discover the functional enrichment of genes with a Ka/Ks ratio greater or equal to 1 [26]. Used GO terms were those previously annotated by Sma3s v2, which extracts these terms from the UniProt entries.

### 2.4. Prediction of Spurious Proteins

Spurio was used to analyze all protein-coding genes in the pangenome, using default parameters [27]. This tool is based on a tblastn search, and it was used to create three different groups of proteins: true proteins (the score that Spurio gives exceeds the default value), no_similarity (Spurio does not find any hit in the similarity search, or the hit does not have a significant e-value), and spurious proteins (the score that Spurio gives does not exceed the default value).

## 3. Results

### 3.1. The Helicobacter Pylori Pangenome Is Twice the Number of Genes of an Independent Genome

**Figure 1:**
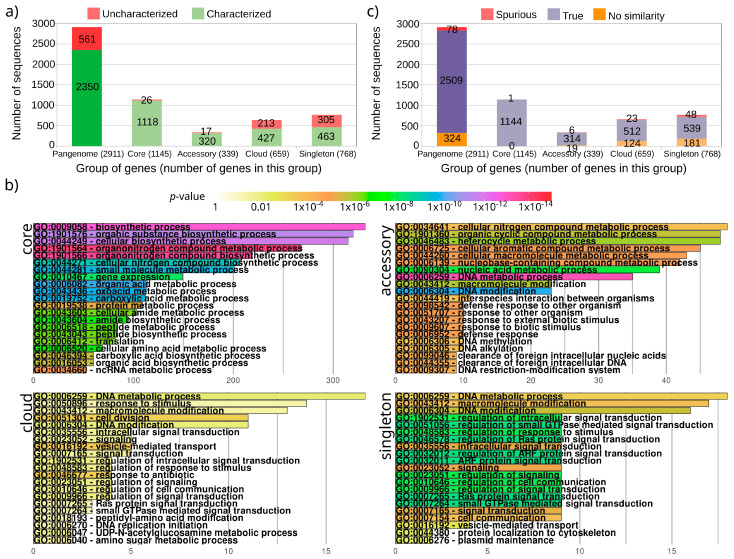
Proteins characterized and uncharacterized in the pangenome and functional enrichment. (**a**) Number of proteins characterized (with annotations) and uncharacterized (without any annotation) in the complete pangenome and in each of its groups. The total number of genes that belong to each group are shown in parentheses. (**b**) Functional enrichment of each group of genes. (**c**) Results of Spurio for proteins in the complete pangenome and in each of its groups: No_similarity (multiple alignment could not be created due to the lack of homologous), True (predicted as true proteins), and Spurious (predicted as spurious proteins).

To calculate the pangenome of *H. pylori*, 180 complete genomes from different strains were used, which presented an average number of 1547 ± 42 protein-coding genes and 38 ± 1 non-coding genes (mainly tRNA), with the 18% of genomes bearing 1–2 plasmids. After the annotation of all the genome sequences, the pangenome was estimated at 2911 protein-coding genes (Supplementary File S1; Supplementary Table S2), with 1145 core genes appearing in all the strains, 768 singleton genes appearing in only one strain, 339 accessory genes (20–99% of strains), and 659 cloud genes (1–20% of genomes). The 19% of proteins encoded by these genes could not be functionally characterized (561 proteins), though these sequences were more abundant in both the singleton and cloud datasets that represent proteins appearing in less than 20% of the strains (Figure 1a).

The core genome is enriched with genes involved in housekeeping tasks and it shows terms such as the biosynthetic process, gene expression, and translation (Figure 1b). Genes in the accessory group were remarkably involved in defense response and cellular aromatic compound metabolism, but also in DNA modifications with processes such as methyltransferase and endonuclease linked to restriction systems used to avoid the entrance of foreign DNA. These systems were distinctive in *H. pylori*, and they have been related to virulence [28,29]. However, the appearance of these functions in the accessory genome proposes that the number of different defense systems in this species vary among strains. For example, some methyltransferases can be used as markers of *H. pylori* geographic distribution [30]. Furthermore, some of these defense systems could be genes involved in CRISPR-Cas systems. Though the presence of this kind of acquired immunity systems is questioned in *H. pylori* [31,32], two of the proteins found in the accessory group were annotated as CRISPR-associated proteins (the singleton *unknown271*, and *mjaIM* that was found in 65 strains and duplicated in some of them; Supplementary Table S2). The cloud group, which includes accessory genes appearing in at least 36 strains, was enriched in regulation as expected, but also in cell division, response to antibiotic, and vesicle-mediated transport. Hence, *H. pylori* is known to have a complex system of protein transport supported by vesicles, which is used to interact with the host cell, and it has also been involved in virulence [17]. Finally, singleton proteins, that could represent genes recently acquired by the species, appear enriched in transduction signal, cell communication, plasmid maintenance, and again vesicle-mediated transport. This enrichment could highlight genes involved in cell communication or interaction with the host, and others could be the genes used by the maintenance of plasmids in the strains that present them. However, these 768 genes could also hide spurious proteins originating from sequencing or assembly errors, as suggested by the high number of uncharacterized proteins found in this group (305 genes accounting for 40% of this group; Figure 1a). In fact, this is the group with a higher number of uncharacterized proteins, followed by the cloud group, while both core and accessory groups only show 43 uncharacterized proteins. Other remarkable proteins in the singleton group are the 16 outer membrane proteins (OMP) that it presents. *H. pylori* has several different families of this kind of proteins with different specializations, including membrane channels of adhesins, which can be an advantage in the special environment where it lives [16]. In fact, the core group showed 32 additional OMPs, while the remaining datasets did not show any additional OMP.

To test if the uncharacterized proteins in the pangenome could be spurious sequences erroneously annotated, all proteins were analyzed with the computational tool Spurio [27], which predicts if the protein can be not true, and it should be removed from the annotation. This tool was used to analyze the coding sequence of each reference in the pangenome. It checks if the homologs of these sequences are free from STOP codons that could point to spurious proteins. So, Spurio was able to analyze 2587 genes and predicted 78 of them as spurious genes putatively not encoding for proteins (Figure 1c). However, the spurious proteins were virtually restricted to those that appear in a low number of strains, with only one protein in the core group and six in the accessory group. Functions of these putative spurious proteins were mainly uncharacterized (29 sequences) or related to membrane location (11 sequences) (Supplementary Table S3). Furthermore, these proteins presented an average length of only 61 ± 38 amino acids. In fact, the core protein that was predicted as spurious had 35 amino acids and only presented the GO term “integral component of membrane”, which reduced the confidence in this sequence.

### 3.2. The Ratio Ka/Ks Using the Pangenome of H. pylori Supports Purifying Selection for Most of the Genes

**Figure 2:**
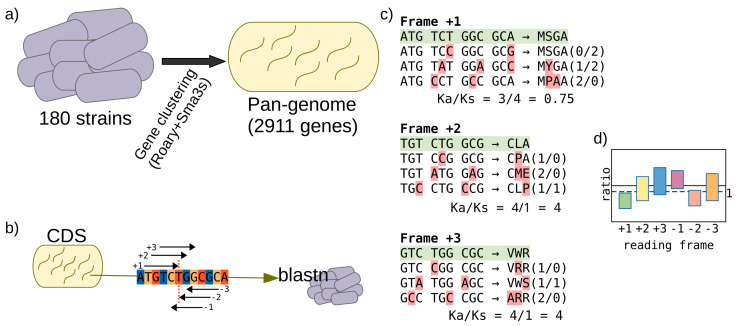
Pangenome details and procedure to calculate the Ka/KS ratio. (**a**) Number of available strains and genes in the pangenome previously obtained. (**b**) The six putative reading frames for each CDS in the pangenome are extracted, and homologous sequences are searched in all the strains (performing a similarity search with BLASTN). Note that the third position in codons from the frame +1 is also the third position of the frame −2 (red dotted line). (**c**) Then, the Ka/Ks for each frame can be calculated starting from the multiple alignments of the homologs from each gene. The initial gene analyzed is highlighted in green, and nucleotide changes are highlighted in red. The number of nonsynonymous and synonymous changes is shown in parentheses. (**d**) Finally, the distribution of Ka/Ks ratios from all the genes in the pangenome can be shown, where we expect a value lower than 1 for most of the genes in the frame +1, and slightly higher values in the frame −2. However, the other four frames should show values greater than 1.

The purifying selection allows protein-coding sequences to conserve the information to translate them into functional proteins. It makes the right reading frame of a coding sequence show a number of synonymous codon changes in a higher number than the nonsynonymous ones. To evaluate this hypothesis with the previously estimated pangenome of *H. pylori*, we calculated the ratio of nonsynonymous (Ka) to synonymous (Ks) substitution rates for all its protein-coding genes (Figure 2). The coding sequence (CDS) of each gene in the pangenome was taken, and a multiple alignment for each of the six reading frames was created. Then, the Ka/Ks ratio was calculated. So, we can compare the obtained ratios, where the frame +1 should show the lower value, being this ideally lower than 1.

**Figure 3:**
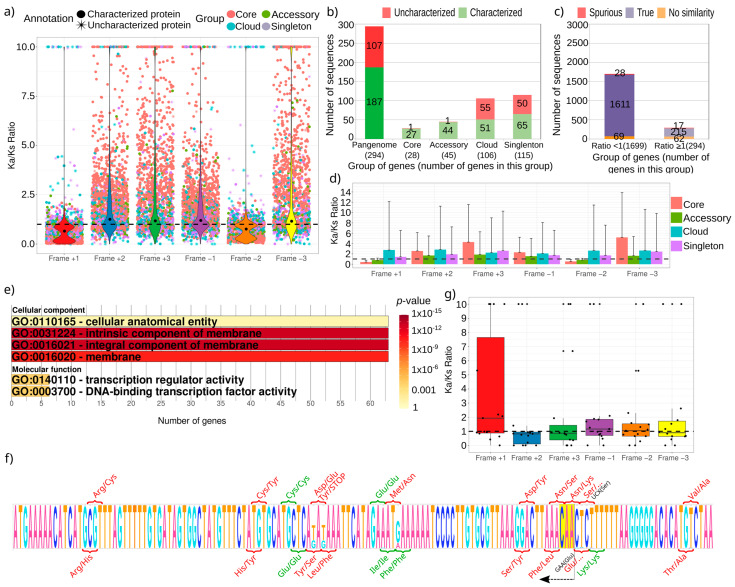
Ka/Ks ratio in the pangenome of *H. pylori*. (**a**) Distribution of the Ka/Ks ratio in all the protein-coding genes from the pangenome, where different groups are highlighted (different colors) and genes that encode for uncharacterized proteins (asterisks). Black dots represent the median of the distribution. Note the double grouping of genes inside both +1 and −2 reading frames. (**b**) Frequency of both characterized and uncharacterized proteins with ratio Ka/Ks greater or equal to 1 separated by the pangenome groups. The total number of genes that belong to each group are shown in parentheses. (**c**) Results of Spurio for proteins with different Ka/Ks ratios: No_similarity (multiple alignment could not be created due to the lack of homologous), True (predicted as true proteins), and Spurious (predicted as spurious proteins). (**d**) Average and standard deviation of the Ka/Ks ratio separated by the different groups and grouped by frames. (**e**) Enrichment of GO terms in the genes with Ka/Ks ratio greater or equal to 1. (**f**) Logo that shows the nucleotide conservation of hpylori124_01049 (gene unknown383) to its homologous genes from all the strains. Above, codons that give amino acid changes in the frame +1 of some strains are shown in green (synonymous) and red (nonsynonymous) colors. Two changes, where the exact amino acid cannot be determined, are shown in black color, with the most probable change in parentheses. Below, the same for frame −2, which uncovers an alternative open reading frame that starts in a TTG codon (CAA in the reverse complementary strand), is highlighted with a yellow background. (**g**) Distribution of the Ka/Ks ratio in the non-coding genes from the pangenome.

As expected, the Ka/Ks ratio presents the lowest value for the frame +1, and most of the genes show a value lower than 1 (Figure 3a). The ratio could be calculated for 80% of genes in the pangenome, due to limitations such as the lack of homologs (2348 out of 2911 genes), though the ratio for the reading frame +1 could only be calculated for 1993 genes, of which 294 showed a ratio greater or equal to 1 (Supplementary Table S3). Most of these latter correspond to genes that did not belong to the core genome (106 genes in the cloud genome and 115 singletons), and more than one third were uncharacterized proteins, with only 2 proteins coming from both the core and the accessory groups (Figure 3b). Therefore, these protein sequences could be encoded by dubious genes or genes that have recently entered the species. In fact, 17 out of these 294 genes were previously predicted as spurious protein-coding genes (Figure 3c). However, some of them correspond to genes with expected selection pressure, such as the proteins involved in virulence *babI*, *cag4*, *cag7*, or OMP proteins *hofB*, *omp22*, *homD*, *hopM*, *fecA2*, and *unknown40* (Table 1).

The remaining reading frames show mainly ratios with high values, except for frame −2 that usually shows a rate lower than 1. This can be explained because this frame shares the third codon position with the frame +1. It makes that these two frames sometimes encode for proteins with overlapping reading frames, as already shown in bacteria [33,34]. Remarkably, the average rate of both frame +1 and frame −2 are lower than 1 in both the core and accessory datasets. However, the other groups, cloud and singleton, present a greater average value with a wide deviation, like that of the other frames (Figure 3d).

To further evaluate the kind of genes that showed a high Ka/Ks ratio, a functional enrichment was performed with those showing a ratio greater than or equal to 1. They mainly were genes encoding to membrane proteins, as most of the proteins involved in virulence or belonging to OMP families, and proteins functioning as transcription regulators (Figure 3e). One of these proteins was hpylori124_01049 (gene unknown383), which presents 8 nonsynonymous and only two synonymous changes (Figure 3f). However, frame −2 present an alternative open reading frame with five nonsynonymous and three synonymous changes that could represent the true reading frame for this gene.

Finally, genes that do not encode proteins should give high Ka/Ks ratios, far from those of frames +1 and −2 from the protein-coding genes. To test this hypothesis, the Ka/Ks ratio was calculated for the non-coding genes of the pangenome, composed of 22 tRNA sequences (Supplementary File S2). As expected, the reading frame +1 showed a high value, but not the remaining frames (Figure 3g). The high value in frame +1 validates that it cannot encode for a protein, but the other frames could support the purifying selection of a protein-coding gene.

## 4. Discussion

The number of sequenced genomes from the same species is growing in the public databases and it enables sequence analyses as never before intended. This allowed us to easily create the pangenome of *H. pylori* that agrees with previous similar estimations that observed it is double of the total number of annotated genes in a single genome, and the core genes comprise up to 75% of them [14,35]. Access to this information allows the evaluation of purifying selection in protein-coding genes starting from intraspecies information, which allows considering a narrower divergence time. So, we show that the results obtained with the 2911 genes in the pangenome of *H. pylori* support the idea that this kind of analyses can be useful to evaluate protein function and helping in sequence annotation procedures. It is known that a protection of the reading frame +1 also protects the frame −2, due to the flexibility of the third position in codons [36]. In fact, some bacteria show overlapping genes involving these reading frames [33,34]. These two frames are also the only ones that show an average Ka/Ks ratio lower than 1, which is shown in our study model, *H. pylori*, with genes appearing in at least 20% of the analyzed strains (Figure 3c). These results were obtained starting from 180 bacterial strains, since only complete genomes were used. However it could be repeated with a greater dataset (around 2000 strains of fragmented genomes of *H. pylori* are now available in the database) in order to validate the conclusions obtained here, and expand the number of analyzed sequences, reaching a greater coverage.

A Ka/Ks ratio greater than 1 could be indicating genes without purifying selection. They could also be uncharacterized genes originating from spurious open reading frames. Hence, we found a high number of these unannotated genes as presenting a high ratio (Figure 3a,b). However, it sometimes suggests that those genes were subjected to high selective pressure, something that is common in pathogenic organisms as studied here. Amino acid changes in proteins encoded by these genes could allow the pathogen to evade the host immune system [37,38]. It has been described in *H. pylori*, and it could explain the great number of analyzed genes with this high ratio [39]. We found that these genes are mainly encoding for membrane protein (Figure 3e), especially outer membrane proteins related to virulence. This fact proposes Ka/Ks with the utility to annotate genes. One example is *omp22*, whose protein is highly immunoreactive and has been proposed as a target for vaccine development [40]. However, the high number of nonsynonymous changes found in this protein predicts an expected divergence of the protein to escape from the vaccine (Table 1).

Another profit of this kind of analysis could be the proposal of uncharacterized proteins as spurious sequences erroneously annotated and stored in databases. We propose 78 spurious proteins from the *H. pylori* pangenome, of which 17 present a Ka/Ks ratio greater or equal to 1, which suggests that they could not be true proteins (Figure 3b), and researchers must decide if these proteins should be considered in other analyses. It is important to highlight here that 62 sequences could not be evaluated as putative spurious genes, so their number could be higher. Since the tool used for finding spurious proteins is based on homologous sequences, these 62 proteins could be reassessed when more sequences were available in the databases.

In addition, the horizontal transfer can affect the results of specific genes when making these studies using prokaryotes, and homoplasy could mask the results. It is particularly important in *H. pylori* due to its highly competent for DNA uptake and recombination [39]. For example, the highly diverse genes *cagA* and *vacA*, which encode for proteins with more than one thousand amino acids in length, could not be analyzed due to their alignments presented long regions with gaps and recombinations [41,42]. In these cases, it is needed to manually repair the initial alignments, removing regions without homology evidence. Despite this, as initially expected, most genes of the pangenome presented purifying selection in the present study.

## 5. Conclusions

In conclusion, studying gene selection using different strain genomes from the same species could be relevant for future computational tools to exhaustively discover the complete set of genes of a genome, and classify these genes. Thus, the use of this information could improve the annotation of whole genomes, for example, by defining improved sequence profiles.

## Figures and Tables

**Table 1:** Outer membrane proteins annotated in the pangenome, together with the Ka/Ks ratio for both +1 and -2 frames, and the group that they belong. The gene name is that annotated by the functional annotator Sma3s. A hyphen symbol appears when the ratio could not be calculated.

Gene	Group	Ratio(+1)	Ratio(-2)	Gene	Group	Ratio(+1)	Ratio(-2)	Gene	Group	Ratio(+1)	Ratio(-2)
alpB	Core	-	-	hopH	Core	0.905		HPAKL86_04155	Singleton	-	-
babB	Singleton	-	-	hopI	Core	0.872	0.939	HPAKL86_05700	Cloud	0.275	0.326
babC	Singleton	-	-	hopK	Core	0.903	1.272	HPY1089_07465	Accessory	0.876	0.870
bamD	Core	0.698	0.857	hopL	Core	0.311	0.893	jhp_0663	Core	0.877	1.065
fecA	Core	-	-	hopM	Singleton	1.001	-	jhp_1360	Core	0.960	
fecA2	Accessory	1.139		hopP	Accessory	-	-	omp13	Singleton	0.125	0.243
frpB	Core	0.732	0.907	hopZ	Accessory	-	-	omp17	Singleton	0.042	0.219
hefD	Core	0.114	0.240	horA	Accessory	-	1.746	omp21	Singleton	0.255	0.389
hefG	Core	0.228	0.334	horB	Core	0.039	0.126	omp22	Singleton	1.225	-
hofB	Core	1.253	1.742	horC	Core	0.124	0.244	omp24	Singleton	0.870	0.967
hofC	Core	0.115	0.233	horE	Core	0.041	0.218	omp25	Singleton	0.922	0.911
hofE	Core	0.370	0.536	horF	Core	0.102	0.182	omp26	Singleton	0.186	0.370
hofF	Core	0.080	0.205	horG	Core	0.238	0.360	omp33	Singleton	0.164	0.302
hofG	Core	0.753	0.889	horH	Core	0.245	0.288	omp4	Singleton	0.316	0.367
homC	Core	-	-	horI	Core	0.210	0.487	omp5	Singleton	-	-
homD	Core	1.010	0.830	horJ	Core	0.125	0.216	ompP1	Core	0.799	0.989
hopA	Core	0.907	0.716	horL	Core	-	1.066	sabA	Singleton	-	-
hopD	Core	0.661		HP_1426	Accessory	0.928	-	sabB	Singleton	0.346	0.537
hopE	Core	0.088	0.132	hp908_0703	Core	0.050	0.195	unknown40	Accessory	1.029	1.009
hopF	Core	0.270	0.352	hp908_1474	Core	0.169	0.273	vlpC	Accessory	-	-

## Data Availability

All data are available in the supplementary files of this article.

## Supplementary Materials

The following are available online at https://www.mdpi.com/2073-4425/12/3/377/s1, Supplementary Table S1. Assemblies downloaded from the NCBI Genome database and used in this article. Supplementary Table S2. Clusters of orthologs with the reference gene in the first column. Each row represents a protein from the pangenome, with the gene name in the first column, the identifier in the second column, and genes considered the same from the remaining strains in the third column. Supplementary File S1. Multifasta with the pangenome protein sequences. Supplementary File S2. Multifasta with the non-coding sequences. Supplementary Table S3. Functional annotation and Ka/Ks ratio for proteins in the pangenome, including non-coding genes, spurious sequences, and those without a calculated ratio.

## References

[1] Mathé C., Sagot M.-F., Schiex T., Rouzé P. (2002). Current Methods of Gene Prediction, Their Strengths and Weaknesses. Nucleic Acids Res..

[2] Park S.J., Son W.S., Lee B.-J. (2012). Structural Analysis of Hypothetical Proteins from Helicobacter Pylori: An Approach to Estimate Functions of Unknown or Hypothetical Proteins. Int. J. Mol. Sci..

[3] Duncan M.C., Gillette R.K., Maglasang M.A., Corn E.A., Tai A.K., Lazinski D.W., Shanks R.M.Q., Kadouri D.E., Camilli A. (2019). High-Throughput Analysis of Gene Function in the Bacterial Predator Bdellovibrio Bacteriovorus. mBio.

[4] Mangas E.L., Rubio A., Álvarez-Marín R., Labrador-Herrera G., Pachón J., Pachón-Ibáñez M.E., Divina F., Pérez-Pulido A.J. (2019). Pangenome of Acinetobacter Baumannii Uncovers Two Groups of Genomes, One of Them with Genes Involved in CRISPR/Cas Defence Systems Associated with the Absence of Plasmids and Exclusive Genes for Biofilm Formation. Microb. Genom..

[5] Sherman R.M., Salzberg S.L. (2020). Pan-Genomics in the Human Genome Era. Nat. Rev. Genet..

[6] Armstrong J., Fiddes I.T., Diekhans M., Paten B. (2019). Whole-Genome Alignment and Comparative Annotation. Annu. Rev. Anim. Biosci..

[7] Oyanedel D., Labreuche Y., Bruto M., Amraoui H., Robino E., Haffner P., Rubio T., Charrière G.M., Le Roux F., Destoumieux-Garzón D. (2020). Vibrio Splendidus O-Antigen Structure: A Trade-off between Virulence to Oysters and Resistance to Grazers. Environ. Microbiol..

[8] Adrian J., Bonsignore P., Hammer S., Frickey T., Hauck C.R. (2019). Adaptation to Host-Specific Bacterial Pathogens Drives Rapid Evolution of a Human Innate Immune Receptor. Curr. Biol..

[9] Weedall G.D., Polley S.D., Conway D.J. (2008). Gene-Specific Signatures of Elevated Non-Synonymous Substitution Rates Correlate Poorly across the Plasmodium Genus. PLoS ONE.

[10] Guéguen L., Duret L. (2018). Unbiased Estimate of Synonymous and Nonsynonymous Substitution Rates with Nonstationary Base Composition. Mol. Biol. Evol..

[11] Jordan I.K., Rogozin I.B., Wolf Y.I., Koonin E.V. (2002). Microevolutionary Genomics of Bacteria. Theor. Popul. Biol..

[12] Algood H.M.S., Cover T.L. (2006). Helicobacter Pylori Persistence: An Overview of Interactions between H. Pylori and Host Immune Defenses. Clin. Microbiol. Rev..

[13] Feldman R.A., Eccersley A.J., Hardie J.M. (1998). Epidemiology of Helicobacter Pylori: Acquisition, Transmission, Population Prevalence and Disease-to-Infection Ratio. Br. Med. Bull..

[14] Gressmann H., Linz B., Ghai R., Pleissner K.-P., Schlapbach R., Yamaoka Y., Kraft C., Suerbaum S., Meyer T.F., Achtman M. (2005). Gain and Loss of Multiple Genes during the Evolution of Helicobacter Pylori. PLoS Genet..

[15] Oleastro M., Ménard A. (2013). The Role of Helicobacter Pylori Outer Membrane Proteins in Adherence and Pathogenesis. Biology.

[16] Alm R.A., Bina J., Andrews B.M., Doig P., Hancock R.E., Trust T.J. (2000). Comparative Genomics of Helicobacter Pylori: Analysis of the Outer Membrane Protein Families. Infect. Immun..

[17] Chmiela M., Walczak N., Rudnicka K. (2018). Helicobacter Pylori Outer Membrane Vesicles Involvement in the Infection Development and Helicobacter Pylori-Related Diseases. J. Biomed. Sci..

[18] Turkina M.V., Olofsson A., Magnusson K.-E., Arnqvist A., Vikström E. (2015). Helicobacter Pylori Vesicles Carrying CagA Localize in the Vicinity of Cell-Cell Contacts and Induce Histone H1 Binding to ATP in Epithelial Cells. FEMS Microbiol. Lett..

[19] Seemann T. (2014). Prokka: Rapid Prokaryotic Genome Annotation. Bioinformatics.

[20] Casimiro-Soriguer C.S., Muñoz-Mérida A., Pérez-Pulido A.J. (2017). Sma3s: A Universal Tool for Easy Functional Annotation of Proteomes and Transcriptomes. Proteomics.

[21] Page A.J., Cummins C.A., Hunt M., Wong V.K., Reuter S., Holden M.T.G., Fookes M., Falush D., Keane J.A., Parkhill J. (2015). Roary: Rapid Large-Scale Prokaryote Pan Genome Analysis. Bioinformatics.

[22] Zhang Z., Li J., Zhao X.-Q., Wang J., Wong G.K.-S., Yu J. (2006). KaKs_Calculator: Calculating Ka and Ks through Model Selection and Model Averaging. Genom. Proteom. Bioinform..

[23] Altschul S.F., Madden T.L., Schäffer A.A., Zhang J., Zhang Z., Miller W., Lipman D.J. (1997). Gapped BLAST and PSI-BLAST: A New Generation of Protein Database Search Programs. Nucleic Acids Res..

[24] Katoh K., Standley D.M. (2013). MAFFT Multiple Sequence Alignment Software Version 7: Improvements in Performance and Usability. Mol. Biol. Evol..

[25] Shen W., Le S., Li Y., Hu F. (2016). SeqKit: A Cross-Platform and Ultrafast Toolkit for FASTA/Q File Manipulation. PLoS ONE.

[26] Alexa A., Rahnenführer J., Lengauer T. (2006). Improved Scoring of Functional Groups from Gene Expression Data by Decorrelating GO Graph Structure. Bioinformatics.

[27] Höps W., Jeffryes M., Bateman A. (2018). Gene Unprediction with Spurio: A Tool to Identify Spurious Protein Sequences. F1000Res.

[28] Humbert O., Salama N.R. (2008). The Helicobacter Pylori HpyAXII Restriction-Modification System Limits Exogenous DNA Uptake by Targeting GTAC Sites but Shows Asymmetric Conservation of the DNA Methyltransferase and Restriction Endonuclease Components. Nucleic Acids Res..

[29] Ando T., Ishiguro K., Watanabe O., Miyake N., Kato T., Hibi S., Mimura S., Nakamura M., Miyahara R., Ohmiya N. (2010). Restriction-Modification Systems May Be Associated with Helicobacter Pylori Virulence. J. Gastroenterol Hepatol..

[30] Vale F.F., Mégraud F., Vítor J.M. (2009). Geographic Distribution of Methyltransferases of Helicobacter Pylori: Evidence of Human Host Population Isolation and Migration. BMC Microbiol..

[31] Bangpanwimon K., Sottisuporn J., Mittraparp-Arthorn P., Ueaphatthanaphanich W., Rattanasupar A., Pourcel C., Vuddhakul V. (2017). CRISPR-like Sequences in Helicobacter Pylori and Application in Genotyping. Gut Pathog..

[32] García-Zea J.A., de la Herrán R., Robles Rodríguez F., Navajas-Pérez R., Ruiz Rejón C. (2019). Detection and Variability Analyses of CRISPR-like Loci in the H. Pylori Genome. PeerJ.

[33] Tunca S., Barreiro C., Coque J.-J.R., Martín J.F. (2009). Two Overlapping Antiparallel Genes Encoding the Iron Regulator DmdR1 and the Adm Proteins Control Siderophore [Correction of Sedephore] and Antibiotic Biosynthesis in Streptomyces Coelicolor A3(2). FEBS J..

[34] Fellner L., Bechtel N., Witting M.A., Simon S., Schmitt-Kopplin P., Keim D., Scherer S., Neuhaus K. (2014). Phenotype of HtgA (MbiA), a Recently Evolved Orphan Gene of Escherichia Coli and Shigella, Completely Overlapping in Antisense to YaaW. FEMS Microbiol. Lett..

[35] Tatusova T., DiCuccio M., Badretdin A., Chetvernin V., Nawrocki E.P., Zaslavsky L., Lomsadze A., Pruitt K.D., Borodovsky M., Ostell J. (2016). NCBI Prokaryotic Genome Annotation Pipeline. Nucleic Acids Res..

[36] Mir K., Schober S. (2014). Selection Pressure in Alternative Reading Frames. PLoS ONE.

[37] Aguileta G., Refrégier G., Yockteng R., Fournier E., Giraud T. (2009). Rapidly Evolving Genes in Pathogens: Methods for Detecting Positive Selection and Examples among Fungi, Bacteria, Viruses and Protists. Infect. Genet. Evol..

[38] Chattopadhyay S., Weissman S.J., Minin V.N., Russo T.A., Dykhuizen D.E., Sokurenko E.V. (2009). High Frequency of Hotspot Mutations in Core Genes of Escherichia Coli Due to Short-Term Positive Selection. Proc. Natl. Acad. Sci. USA.

[39] Chattopadhyay S., Chi P.B., Minin V.N., Berg D.E., Sokurenko E.V. (2018). Recombination-Independent Rapid Convergent Evolution of the Gastric Pathogen Helicobacter Pylori. BMC Genom..

[40] Kim J.S., Chang J.H., Seo W.Y., Yu G.J., Chung S.I., Yum J.S. (2000). Cloning and Characterization of a 22 KDa Outer-Membrane Protein (Omp22) from Helicobacter Pylori. Mol. Cells.

[41] López-Vidal Y., Ponce-de-León S., Castillo-Rojas G., Barreto-Zúñiga R., Torre-Delgadillo A. (2008). High Diversity of VacA and CagA Helicobacter Pylori Genotypes in Patients with and without Gastric Cancer. PLoS ONE.

[42] Yamazaki S., Yamakawa A., Okuda T., Ohtani M., Suto H., Ito Y., Yamazaki Y., Keida Y., Higashi H., Hatakeyama M. (2005). Distinct Diversity of VacA, CagA, and CagE Genes of Helicobacter Pylori Associated with Peptic Ulcer in Japan. J. Clin. Microbiol..

